# Wegen Anorexia nervosa auf die Palliativstation?

**DOI:** 10.1007/s00115-023-01498-0

**Published:** 2023-05-30

**Authors:** Sascha Weber, Michael Paulzen, Frank Elsner, Sandra Weeger-Elsner, Dominik Groß, Manuel Trachsel, Roman Rolke, Anna L. Westermair

**Affiliations:** 1 Klinik für Palliativmedizin, Medizinische Fakultät, RWTH Aachen University, Aachen, Deutschland; 2 Klinik für Psychiatrie, Psychotherapie und Psychosomatik, Medizinische Fakultät, RWTH Aachen University, Aachen, Deutschland; 3 Alexianer Krankenhaus, Aachen, Deutschland; 4 Rechtsanwaltskanzlei Weeger-Elsner, Köln, Deutschland; 5 Institut für Geschichte, Theorie und Ethik der Medizin, Medizinische Fakultät, RWTH Aachen University, Aachen, Deutschland; 6 Abteilung Klinische Ethik, Universitätsspital Basel (USB), Universitäre Psychiatrische Kliniken (UPK), Universitäre Altersmedizin Felix-Platter (UAFP), Universitäres Kinderspital beider Basel (UKBB), Spitalstrasse 21, 4031 Basel, Schweiz; 7 Institut für Biomedizinische Ethik und Geschichte der Medizin, Universität Zürich, Zürich, Schweiz

Mit der vorliegenden Fallbeschreibung möchten wir eine Diskussion über den Einsatz palliativmedizinischer Ansätze bei besonders schweren Ausprägungen psychischer Erkrankungen anregen.

## Fallbeschreibung

**Figure 1:**
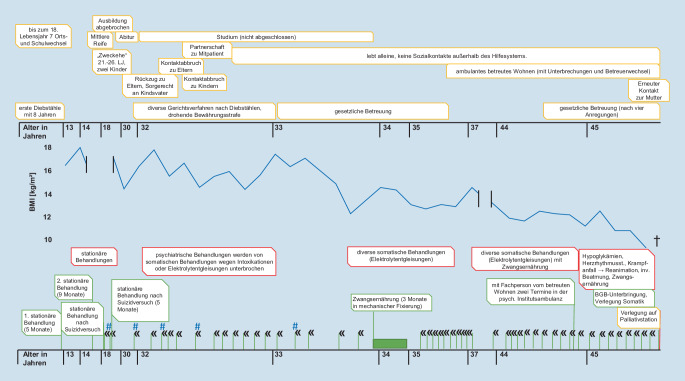

Die 45-jährige Patientin litt seit über 30 Jahren an einer Anorexia nervosa (AN) vom restriktiven Typ, einer kombinierten Persönlichkeitsstörung mit emotional-instabilen und dissozialen Anteilen und einem schädlichen Gebrauch von Benzodiazepinen. Die Behandlungs- und Lebensgeschichte (Abb. 1) war geprägt von Beziehungsabbrüchen im persönlichen wie professionellen Bereich, rezidivierender Suizidalität und Suizidversuchen sowie somatischen Komplikationen der AN, Zwangsmaßnahmen und irregulären Behandlungsbeendigungen. Mehrfach wurde sie durch Diebstähle und Brandstiftung straffällig.

In über 60 stationären psychiatrischen Behandlungen in 8 z. T. hoch spezialisierten Einrichtungen konnten keine (bleibenden) Erfolge über die Lebenserhaltung hinaus erreicht werden. Selbst unter einer 12-monatigen Zwangsernährung, davon mehrere Monate in mechanischer Fixierung, sank ihr Gewicht. Wiederholte Angebote einer ambulanten psychiatrischen und psychotherapeutischen Behandlung wurden nur sehr vereinzelt wahrgenommen.

In den letzten Jahren hatte die Patientin ihr Leben durchgehend als nicht mehr lebenswert betrachtet und dies mehrfach gegenüber ihren Behandlungsteams geäußert.

Im Rahmen einer freiheitsentziehenden Unterbringung nach dem Betreuungsrecht wegen Eigengefährdung durch progredienten Gewichtsverlust erlitt die Patientin schließlich einen Krampfanfall, wahrscheinlich als Folge von Hypoglykämie, mit respiratorischer Insuffizienz und Herzrhythmusstörungen. Sie wurde reanimiert, invasiv beatmet und anschließend parenteral ernährt. Nach Wiedererlangen des Bewusstseins lehnte die Patientin die Fortführung der künstlichen Ernährung strikt ab. Für diese Entscheidung wurde sie wegen extremer Angst vor Gewichtszunahme von zwei unabhängigen Fachärzt*innen für Psychiatrie als einwilligungsunfähig eingeschätzt.

### Psychiatrische Perspektive.

Nach übereinstimmender Einschätzung durch mehrere unabhängige Fachärzt*innen war anzunehmen, dass die Patientin im Verlauf nicht die notwendige Kooperation würde zeigen können, um von einer Behandlung ihrer AN zu profitieren. Ein letztlich letaler Verlauf schien mit und ohne Zwangsernährung hochwahrscheinlich. Entsprechende negative Prädiktoren waren der extrem niedrige BMI, die Komorbiditäten, die lange Krankheitsdauer von über 30 Jahren, das höhere Alter, das niedrige psychosoziale Funktionsniveau, die hohe Symptombelastung, die fehlende Therapiemotivation, die häufigen Hospitalisierungen, die wiederholten und lange aufrechterhaltenen Zwangsmaßnahmen und die irregulären Behandlungsbeendigungen [2, 3, 5–7].

### Ethische Perspektive.

Die Patientin war als nicht einwilligungsfähig eingeschätzt worden und hatte keinen Willen vorausverfügt. Die früheren Ablehnungen künstlicher Ernährung sowie die subjektiv inakzeptable Lebensqualität könnten darauf hindeuten, dass eine weitere künstliche Ernährung nicht im Sinn der Patientin wäre [9]. Da die Patientin jedoch wahrscheinlich auch bei den früheren Ablehnungen einwilligungsunfähig war, sind diese Indizien auf den mutmaßlichen Patientenwillen nicht stichhaltig. Also war das medizinethische Prinzip des Respekts vor der Autonomie [1] im vorliegenden Fall nicht handlungsleitend (s. [4] für eine Diskussion bei einwilligungsfähigen Patient*innen). Deswegen war auf das objektiv beste Interesse der Patientin abzustellen. Dabei bot das Prinzip des Wohltuns (Benefizienz) kaum Unterstützung für eine Zwangsernährung: Die Lebensqualität war auch vor der letzten Verschlechterung subjektiv inakzeptabel gewesen, eine Besserung der AN und damit der Lebensqualität war aller Voraussicht nach nicht erreichbar und ein letaler Verlauf auch mit Zwangsernährung wahrscheinlich. Zu einem ähnlichen Resultat führt die Überprüfung des Prinzip des Nichtschadens (Nonmalefizienz): Die zu erwartende hohe psychische Belastung und das Komplikationsrisiko sprachen gegen eine Zwangsernährung. Bei anhaltend inakzeptabler Lebensqualität ohne realistische Aussichten auf Besserung hätten lebenserhaltende Maßnahmen aller Voraussicht nach lediglich einen leidvollen Zustand verlängert, was für ein Unterlassen sprach.

### Rechtliche Perspektive.

Unter medikolegalen Gesichtspunkten werden der Verlauf und Sachverhalt der Patientin gut durch die Begrifflichkeit und Diskussion des *Sterben Zulassens* (einem Teilaspekt des früher angewandten Begriffs der passiven Sterbehilfe) beschrieben: In Abgrenzung zur strafbaren *Tötung auf Verlangen* (§ 216 StGB; früher als aktive Sterbehilfe bezeichnet) wird passive Sterbehilfe im Sinne einer Nichtaufnahme oder Beendigung lebensverlängernder Maßnahmen bei schwerkranken Patient*innen strafrechtlich nicht geahndet, wenn keine Indikation (mehr) für die medizinischen Maßnahmen besteht und/oder dies dem erklärten oder mutmaßlichen Willen der Patient*innen entspricht (vgl. BGH Urteil vom 25.06.2010, AZ: 2 StR 454/09). In Anlehnung an die ethische Perspektive wurde für die Patientin keine Indikation für eine Zwangsernährung mehr gesehen. Mit einer strafrechtlichen Verurteilung des beschriebenen Vorgehens ist nicht zu rechnen.

### Verlauf.

Aufgrund der Komplexität des Falls wurden parallel eine Klinische Ethikberatung einberufen und eine Begutachtung bezüglich weiterer Unterbringung und Zwangsbehandlung beim Betreuungsgericht beantragt. Den Empfehlungen der Klinischen Ethikberatung folgend entschieden die Behandelnden zusammen mit der gesetzlichen Betreuerin und der forensisch-psychiatrischen Gutachterin gegen eine weitere Zwangsernährung (wegen des ungünstigen Nutzen-Schaden-Verhältnisses) und für eine Verlegung auf die Palliativstation. Die Patientin war mit diesem Prozedere einverstanden. Auf der Palliativstation konnten Symptome wie Schmerzen und Luftnot gelindert und die Patientin dabei unterstützt werden, wieder Kontakt mit ihrer Mutter aufzunehmen. Sie verstarb neun Tage später.

### Einordnung.

Außerhalb Deutschlands wurden bereits mehrere ähnliche Fälle publiziert, in denen auf lebensrettende Zwangsmaßnahmen bei AN verzichtet wurde, weil dies als im besten Interesse der einwilligungsunfähigen Patient*innen angesehen wurde (für einen Überblick s. [8]). Anekdotische Evidenz deutet darauf hin, dass der hier vorgestellte Fall auch in Deutschland kein Einzelfall ist. Jedoch fehlen entsprechende epidemiologische Untersuchungen, Konsenskriterien für den Verzicht auf Zwangsmaßnahmen trotz vitaler Gefährdung sowie Konzepte und Protokolle für die Sterbebegleitung bei schwerstgradig ausgeprägter AN. Deren Entwicklung wurde von international renommierten AN-Expert*innen gefordert [10].

## Fazit für die Praxis

- In besonders schweren Fällen von AN kann die Lebensqualität derart schlecht und die Aussicht auf Besserung derart gering sein, dass ein Zulassen des Versterbens im Rahmen einer spezialisierten Palliativversorgung die am wenigsten schlechte Option darstellen könnte.
- Interdisziplinäre Abstimmung mit Klinischer Ethik, Betreuungsgericht und den Betroffenen bzw. ihren gesetzlichen Betreuer*innen ist nötig, um ein medizinisch wie auch ethisch rechtfertigbares Prozedere zu finden.
